# REFINE: a database of linked clinical data and genomic biomarkers in renal cell carcinoma patients receiving immunotherapy-based treatment regimens

**DOI:** 10.1093/oncolo/oyaf135

**Published:** 2025-06-19

**Authors:** Jeffrey Zhong, Albert Jang, Bashar Abuqayas, Arnab Basu, David J Benjamin, Vineel Bhatlapenumarthi, Mehmet Asim Bilen, Dhvani Buch, Mark Chang, Erica Chin, Sourat Darabi, Nagendra Dhanikonda, Pooja Ghatalia, Claud M Grigg, Abby L Grier, Tanya Jindal, Joannah Jung, Deepak Kilari, Hamsa L S Kumar, Suzanna Lee, Brittany Neelands, Chinmayi Pandya, Jeff Pawelek, Jaimee Staggers, Ahmet Yildirim, Yousef Zakharia, Kevin K Zarrabi, Michael Zimmerman, George Sledge, David Spetzler, Andrew Elliott, Rana R McKay, Pedro C Barata

**Affiliations:** Division of Solid Tumor Oncology, Department of Medicine, University Hospitals Cleveland Medical Center, Case Western Reserve University School of Medicine, Cleveland, OH 44106, United States; Division of Solid Tumor Oncology, Department of Medicine, University Hospitals Cleveland Medical Center, Case Western Reserve University School of Medicine, Cleveland, OH 44106, United States; Division of Oncology, Holden Comprehensive Cancer Center, Iowa City, IA 52242, United States; Division of Hematology-Oncology, University of Alabama O’Neal Comprehensive Cancer Center, Birmingham, AL 35233, United States; Hoag Family Cancer Institute, Newport Beach, CA 92663, United States; Division of Oncology, Medical College of Wisconsin, Milwaukee, WI 53226, United States; Genitourinary Medical Oncology Program, Department of Hematology and Medical Oncology, Winship Cancer Institute of Emory University, Atlanta, GA 30322, United States; Department of Solid Tumor Oncology, Atrium Health Wake Forest Baptist Comprehensive Cancer Center, Charlotte 28204, United States; Department of Solid Tumor Oncology, Atrium Health Wake Forest Baptist Comprehensive Cancer Center, Charlotte 28204, United States; Division of Hematology-Oncology, University of California San Diego Moores Cancer Center, La Jolla, CA 92307, United States; Hoag Family Cancer Institute, Newport Beach, CA 92663, United States; Department of Hematology-Oncology, Fox Chase Cancer Center, Philadelphia, PA 19111, United States; Department of Hematology-Oncology, Fox Chase Cancer Center, Philadelphia, PA 19111, United States; Department of Solid Tumor Oncology, Atrium Health Wake Forest Baptist Comprehensive Cancer Center, Charlotte 28204, United States; Division of Solid Tumor Oncology, Department of Medicine, University Hospitals Cleveland Medical Center, Case Western Reserve University School of Medicine, Cleveland, OH 44106, United States; Division of Solid Tumor Oncology, Department of Medicine, University Hospitals Cleveland Medical Center, Case Western Reserve University School of Medicine, Cleveland, OH 44106, United States; Division of Hematology-Oncology, University of California San Diego Moores Cancer Center, La Jolla, CA 92307, United States; Division of Oncology, Medical College of Wisconsin, Milwaukee, WI 53226, United States; Division of Solid Tumor Oncology, Department of Medicine, University Hospitals Cleveland Medical Center, Case Western Reserve University School of Medicine, Cleveland, OH 44106, United States; Division of Hematology-Oncology, University of California San Diego Moores Cancer Center, La Jolla, CA 92307, United States; Department of Solid Tumor Oncology, Atrium Health Wake Forest Baptist Comprehensive Cancer Center, Charlotte 28204, United States; Division of Hematology-Oncology, University of California San Diego Moores Cancer Center, La Jolla, CA 92307, United States; Division of Hematology-Oncology, University of California San Diego Moores Cancer Center, La Jolla, CA 92307, United States; Division of Hematology-Oncology, University of Alabama O’Neal Comprehensive Cancer Center, Birmingham, AL 35233, United States; Genitourinary Medical Oncology Program, Department of Hematology and Medical Oncology, Winship Cancer Institute of Emory University, Atlanta, GA 30322, United States; Division of Oncology, Holden Comprehensive Cancer Center, Iowa City, IA 52242, United States; Department of Medical Oncology, Thomas Jefferson Sidney Kimmel Cancer Center, Philadelphia, PA 19107, United States; Department of Medical Oncology, Thomas Jefferson Sidney Kimmel Cancer Center, Philadelphia, PA 19107, United States; Caris Life Sciences, Irving, TX 75039, United States; Caris Life Sciences, Irving, TX 75039, United States; Caris Life Sciences, Irving, TX 75039, United States; Division of Hematology-Oncology, University of California San Diego Moores Cancer Center, La Jolla, CA 92307, United States; Division of Solid Tumor Oncology, Department of Medicine, University Hospitals Cleveland Medical Center, Case Western Reserve University School of Medicine, Cleveland, OH 44106, United States

**Keywords:** renal cell carcinoma, immunotherapy, biomarkers, molecular sequencing

## Abstract

The REnal cancer consortium for Focused Investigation of Novel biomarkers and Expression (REFINE) consortium represents an important initiative in integrating clinical data with molecular sequencing in patients with advanced renal cell carcinoma (RCC) treated with immunotherapy-based approaches. By leveraging real-world evidence and genomic analysis, this consortium aims to explore putative predictive biomarkers with the potential to inform personalized treatment strategies. Findings from the REFINE database may further contribute to our understanding of disease courses of immunotherapy-based approaches for various molecular subtypes of RCC, associations of race and ethnicity with RCC treatment and outcomes with representation of patient populations underrepresented in clinical trials, and optimizing treatment sequencing.

## Introduction

Immunotherapy-based regimens have become the standard of care for metastatic renal cell carcinoma (mRCC), particularly in the treatment of clear cell mRCC.^1,2^ Renal cell carcinoma of variant histology (vRCC, ie, nonclear cell histologies) encompasses different diseases with different biology, clinical behavior, and treatment options including several options including TKI monotherapy, ICI-TKI, ICI-ICI, and rarely chemotherapy.^3^ Despite these observed improvements in clinical outcomes, vast majority of patients either develop acquired or have intrinsic resistance to ICI-based treatment regimens.^4^ To date, no genomic biomarkers currently validated are used clinically to guide selection of ICI-based treatments for advanced RCC; however, several clinical trials (ie, IMmotion 151, JAVELIN Renal 101, Checkmate-214, CLEAR, Keynote-426, and BIONIKK) that examined genomic signatures and their associations with treatment outcomes have been promising.^5–10^ Further identification of these and other putative biomarkers to guide the selection of immunotherapy-based regimens is an unmet clinical need.

Clinical trials fail to capture the diversity of treatment of metastatic RCC. Indeed, racially and ethnically diverse populations are not well represented in studies examining molecular alterations and their associations with treatment outcomes in metastatic RCC. Furthermore, real-world data can help to inform the performance and tolerability of these immunotherapy-based regimens in practice and in a broader diverse population of patients. The REnal cancer consortium for Focused Investigation of Novel biomarkers and Expression (REFINE) is a consortium of several U.S. academic institutions with the shared goal of improving the clinico-genomic characterization of advanced RCC in patients treated with immunotherapy-based regimens using real-world data. Herein, we review the objectives and design of the REFINE consortium.

## Development of the REFINE consortium

The REFINE consortium, initially formed in September 2023, consists of 12 U.S. academic institutions dedicated to further investigation of the real-world clinical and genomic features of patients with advanced RCC treated with immunotherapy-based approaches (Table 1). This consortium aims to expand to other U.S. academic institutions. The primary aims of this consortium are to: (1) explore predictive biomarkers (such as individual gene alterations, as well as broader genomic signatures and expression profiles) and their associations with clinical outcomes to different immunotherapy-based regimens, including OS, PFS, and ORR, and (2) examine the efficacy and tolerability of immunotherapy-based regimens in various settings in patients with previously untreated advanced RCC using real-world data. Secondary aims include exploring other clinically meaningful outcomes, such as those related to management of adverse events, dose modifications and interruptions, use of immunosuppressive medications, and hospitalizations.

**Table 1.** Current REFINE consortium participating institutions.

REFINE Participating Institutions	
Institution Name	Location
Atrium Health Wake Forest Baptist Comprehensive Cancer Center	Charlotte, North Carolina
Fox Chase Cancer Center	Philadelphia, Pennsylvania
Medical College of Wisconsin	Milwaukee, Wisconsin
Hoag Family Cancer Institute	Newport Beach, California
Holden Comprehensive Cancer Center	Iowa City, Iowa
Thomas Jefferson Sidney Kimmel Cancer Center	Philadelphia, Pennsylvania
University of Alabama O’Neal Comprehensive Cancer Center	Birmingham, Alabama
University of California San Diego Moores Cancer Center	La Jolla, California
University Hospitals Seidman Cancer Center	Cleveland, Ohio
Winship Cancer Institute of Emory University	Atlanta, Georgia

Inclusion criteria for patients in this database include: (1) male or female ≥18 years of age, (2) histologically confirmed RCC, either clear cell or RCC of variant histology, (3) treatment naïve for locally advanced or metastatic disease at time of starting immunotherapy-based treatment (treatment of nonmetastatic disease is allowed), (4) subjects have received ≥1 dose of an ICI at the time of study entry, (5) metastatic disease documented on any imaging modality, and (6) available tissue confirming RCC with tissue-based molecular sequencing performed by commercially available CLIA-certified lab (Caris Life Sciences). Using the same methodology via Caris Life Sciences for genomic sequencing at all sites will optimize consistency, reliability, and ease of access to genomic data. Exclusion criteria for patients in this database include: (1) patients without sufficient tissue available for molecular profiling, (2) patients with other advanced (stage 4) cancers, and (3) patients without available clinical data on front-line ICI-based regimen. Variables of interest include tumor histologic characteristics, genomic data, patient and disease characteristics, and treatment characteristics (Table 2). All data will be deidentified, collected, and stored in a secure REDCap database without any protected health information. Each patient entry included in this analysis must have all data related to clinical outcomes and genomic sequencing available in this archive.

**Table 2.** Clinical and genomic variables of interest in REFINE consortium.

Demographics	Histological characteristics	Next-generation sequencing	Patient and disease characteristics	Treatment characteristics
Age	Histology	Whole genome sequencing	History of nephrectomy	Treatment setting (neoadjuvant, adjuvant, for irresectable/locally advanced/metastatic disease)
Race/Ethnicity	Fuhrman grade	Whole transcriptome sequencing	IMDC risk factors	Systemic therapies
Distance from cancer center	Sarcomatoid and rhabdoid features		Karnofsky and ECOG performance status	Starting dose
Smoking status	Pathological staging at diagnosis		Sites of metastases	Duration of therapy
			Weight at diagnosis and start of treatments	Radiographic response
			Genomic analysis including whole exosome sequencing, whole transcriptome sequencing, and immunohistochemistry	Best response
				Dose reductions and discontinuations
				Therapy toxicities
				Requirement of steroids or other immunosuppressive agents
				Hospitalizations

As of December 31st, 2024, the first 150 patients (71% male, 65% ccRCC, and 35% vRCC, 73% with metastatic disease) were included. Among those treated with front-line immunotherapy-based regimens, 59% received TKI-IO, 33% IO-IO, and 8% IO monotherapy. Future goals include having 250 patients added to the database by December 31st, 2025.

## Future directions

Within a short period of time, we have made progress toward gathering large amounts of patient clinical and genomic information that can be used to answer important clinical questions related to the treatment of advanced RCC. We are continuing to expand the database and add additional contributing academic institutions to make the REFINE consortium more robust and its findings more generalizable. As this consortium aims to collect a large amount of clinical data, our vision is for this database to be utilized to pursue additional projects with broad ranges of topics other than investigating genomic signatures. The REFINE consortium is also an opportunity for mentorship of junior investigators and trainees, and for building connections among RCC investigators and physicians to form a basis of future RCC scholarship and collaboration.

## Conclusion

Recent investigations of genomic signatures in the setting of advanced RCC have shown promise for the future of precision oncology. Increasingly individualized treatment of advanced RCC is a growing possibility, especially with regard to optimizing choice of immunotherapy regimens for this heterogenous disease with subset stratification of biomarker analyses. The REFINE consortium aims to link clinical and genomic data for patients with advanced RCC being treated with immunotherapy-based approaches for the investigation of novel biomarkers and study of various aspects of diverse immunotherapy treatment regimens, with all next-generation sequencing performed via the same methodology. This crosstalk between retrospective and prospective data can enhance the investigation of predictive biomarkers and their clinical associations in patients with advanced RCC, assisting with further development of individualized treatment for molecular subsets of patients. We hope that this consortium will be able to provide answers for important clinical questions and eventually improve outcomes for patients with advanced RCC.

## Data Availability

The data underlying this article were provided by Caris Life Sciences under license/by permission. Data will be shared on request to the corresponding author with permission of Caris Life Sciences.
