# Correction to: REFINE: a database of linked clinical data and genomic biomarkers in renal cell carcinoma patients receiving immunotherapy-based treatment regimens

**DOI:** 10.1093/oncolo/oyaf302

**Published:** 2025-10-13

This is a correction to: Jeffrey Zhong, Albert Jang, Bashar Abuqayas, Arnab Basu, David J Benjamin, Vineel Bhatlapenumarthi, Mehmet Asim Bilen, Dhvani Buch, Mark Chang, Erica Chin, Sourat Darabi, Nagendra Dhanikonda, Pooja Ghatalia, Claud M Grigg, Abby L Grier, Tanya Jindal, Joannah Jung, Deepak Kilari, Hamsa L S Kumar, Suzanna Lee, Brittany Neelands, Chinmayi Pandya, Jeff Pawelek, Jaimee Staggers, Ahmet Yildirim, Yousef Zakharia, Kevin K Zarrabi, Michael Zimmerman, George Sledge, David Spetzler, Andrew Elliott, Rana R McKay, Pedro C Barata, REFINE: a database of linked clinical data and genomic biomarkers in renal cell carcinoma patients receiving immunotherapy-based treatment regimens, *The Oncologist*, Volume 30, Issue 6, June 2025, oyaf135, https://doi.org/10.1093/oncolo/oyaf135

In the originally published version of this manuscript, a typographical error was present in author Jeff Pawelek’s name.

This has been corrected.

